# Bivalve Gut Microbiome Responses to Sediment Characteristics and Anthropogenic Activities in *Cerastoderma edule* (Linnaeus, 1758)

**DOI:** 10.1007/s00248-026-02847-7

**Published:** 2026-08-15

**Authors:** Joana M. Neves, Mário Mil-Homens, Marta Martins, Helena Adão, Sara Gutierrez-Patricio, Pedro Nolasco Jiménez, Jorge R. Osman, Ana Z. Miller, Jorge Lobo-Arteaga

**Affiliations:** 1 MARE - Marine and Environmental Sciences Centre, ARNET - Aquatic Research Network Associate Laboratory, Department of Environmental Sciences and Engineering, NOVA School of Science and Technology, NOVA University Lisbon, Caparica, Portugal; 2 IPMA – Portuguese Institute for Sea and Atmosphere, I.P., Av. Alfredo Magalhães Ramalho 6, 1495-165 Algés, Portugal; 3 MARE – Marine and Environmental Sciences Centre/ARNET - Aquatic Research Network, Universidade de Évora, Escola de Ciências E Tecnologia, Apartado 94, 7002-554 Évora, Portugal; 4 HERCULES Laboratory, University of Évora, Largo Marquês de Marialva 8, 7000-809 Évora, Portugal; 5 BIOGEOCOM Research Group, Instituto de Recursos Naturales y Agrobiología de Sevilla – Consejo Superior de Investigaciones Científicas (IRNAS-CSIC), Av. Reina Mercedes 10, 41012 Seville, Spain; 6 CIIMAR/CIMAR-LA, Centro Interdisciplinar de Investigacao Marinha E Ambiental, Universidade Do Porto, Terminal de Cruzeiros de Leixões, Av. General Norton de Matos S/N, 4450-208 Matosinhos, Portugal

**Keywords:** Environmental disturbance, 16S rRNA gene, ITS sequencing, Estuarine ecotoxicology, Host–microbe interactions, Functional prediction

## Abstract

Estuaries are dynamic ecosystems influenced by fluctuating environmental conditions and sediment-associated contamination, which may affect host-associated microbiomes. This study investigated the gut microbiome of the bivalve *Cerastoderma edule* across three estuarine sites differing in anthropogenic pressure. Bacterial 16S rRNA and fungal ITS metabarcoding data were integrated with sediment physicochemical parameters to assess spatial and seasonal variation in gut microbial diversity and composition. Bacterial communities showed clear differences among sites and seasons, with stronger variation in areas characterized by higher organic matter and trace metal concentrations. Fungal communities also varied among samples, although their interpretation was limited by the high proportion of unassigned ITS taxa. Multivariate analyses indicated associations between gut microbial composition and sediment parameters, including organic matter, salinity, and trace metals such as mercury (Hg), zinc (Zn), and lead (Pb). Putative bacterial functional profiles were inferred from taxonomic data using FAPROTAX, suggesting potential involvement in nitrogen, sulfur, and carbon cycling, as well as organic compound degradation. Nevertheless, it is important to note that these functional predictions should be interpreted as indicators of potential metabolic capacity rather than direct evidence of activity. Ultimately, these findings demonstrate that the *C. edule* gut microbiome has strong potential as a complementary tool for the assessment of estuarine environmental conditions and host–environment interactions.

**Supplementary Information:**

The online version contains supplementary material available at https://doi.org/10.1007/s00248-026-02847-7.

## Introduction

Microbiomes, defined as communities of microorganisms, such as bacteria, archaea, fungi and viruses, that inhabit a given environment or host, are increasingly recognized as indicators of the host condition and ecosystem health [1–4], playing an important role in digestion [5], metabolism [6], immune system regulation [7], nutrient cycling and organic matter decomposition [8]. Marine benthic macro- and micro-organisms are intrinsically linked and work together to facilitate essential physical and chemical processes, including the carbon and nitrogen cycles, which directly influence sediment quality and the associated infaunal communities [9, 10]. Bivalves host diverse microbial communities that are shaped by both intrinsic host-related factors and extrinsic environmental conditions [11], such as temperature, salinity, seasonal variability and contamination [12, 13]. These characteristics underscore the microbiome's potential as a valuable sentinel in ecological monitoring [13, 14]. However, although bacterial communities have been the focus of extensive research, fungal communities of the bivalve microbiome remain comparatively underexplored, despite growing evidence that marine fungi contribute to ecosystem functioning, host health and potential interactions with bacterial communities [15, 15–19]. A more comprehensive and fundamental understanding of how benthic host-associated bacterial and fungal communities respond to environmental stressors is therefore essential for improving assessments of anthropogenic impacts on marine ecosystems.

Environmental conditions, including salinity, temperature, and organic and inorganic contaminants, profoundly influence the microbiome community structure and functionality [20, 21]. Research on oysters (*Crassostera* spp.) and mussels (*Mytilus* spp.) has demonstrated the impact of these parameters on microbiome composition in different organs, influencing not only the health of the host organism but also the mortality rates and aquaculture profitability [13, 22]. Furthermore, anthropogenic contaminants, including trace metals and petroleum-derived compounds, can alter gut-associated microbial communities, highlighting their sensitivity to environmental disturbances [2, 4, 14, 23–26]. The common cockle (*Cerastoderma edule (Linnaeus, 1758)*) is of considerable economic and ecological importance. In Portugal, it is a commercially valuable species, widely harvested for human consumption and an important component of estuarine ecosystems. As a suspension-feeding and sediment-associated bivalve, *C. edule* contributes to nutrient cycling, links benthic and pelagic processes, and serves as prey for birds and other animals [27, 27, 28]. Their recognized status as bioindicators, their ecological role and their interactions with parasites, sediment microbiota, and environmental factors, suggest a complex microbial association [29, 30]. Although studies specifically on the gut microbiome of *C. edule* are still lacking, its biological and ecological characteristics make it a highly relevant modelspecies for investigating host–microbiome interactions in estuarine environments. This is further supported by research on other bivalves in aquaculture, which highlights the importance of studying gut microbiomes in natural populations to better understand resilience to environmental stressors [31].

This study aims to assess the influence of anthropogenic pressure on the gut microbiome of *C. edule* across the Sado Estuary, Portugal. Using next-generation sequencing (NGS), we characterized both bacterial and fungal communities and linked microbiome patterns to sediment physicochemical characteristics, environmental parameters, and trace-metal concentrations across two contrasting seasons.

## Materials and Methods

### Study Area

The Sado estuary, located in the southwestern Portuguese coast, follows a warm-temperate, semi-diurnal mesotidal regime, with tidal ranges between 0.6 m during neap and 1.6 m during spring tides [32]. The intertidal zones of the estuary cover approximately ~ 78 km^2^, with salt marshes and intertidal plains making up about 30% of the area [33]. It connects to the ocean through a narrow, deep channel (up to 50 m deep) and is partially divided by intertidal sandbanks. The river inflow fluctuates seasonally, with most freshwater discharges occurring in winter [34].

**Fig. 1:**
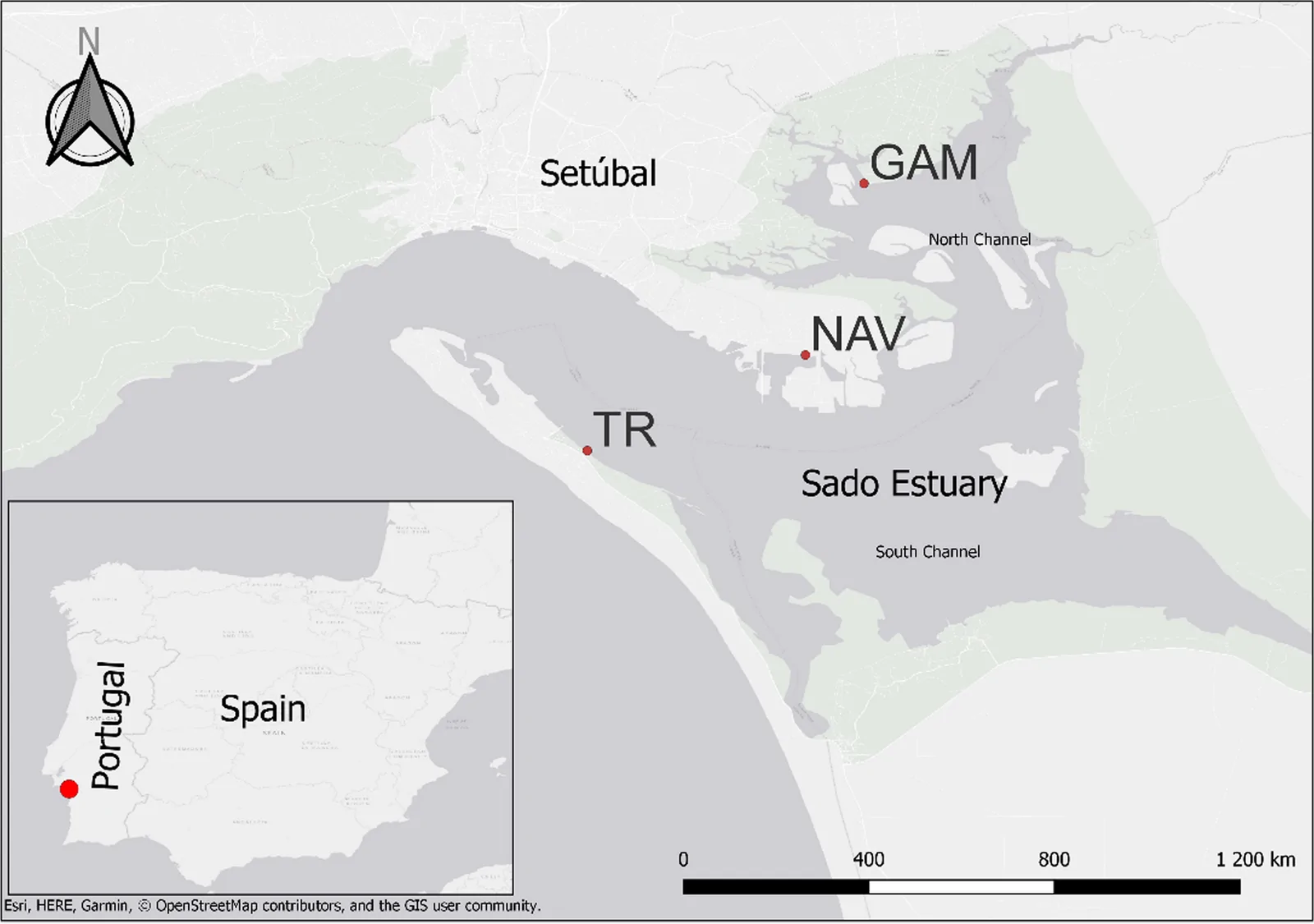
Location of the 3 sampling sites in Sado Estuary: Tróia (TR); Navigator (NAV); Gâmbia (GAM)

Three distinct areas were selected, based on their biogeochemistry and adjacent anthropogenic activities [35]: Tróia (TR) in the South Channel, and Gâmbia (GAM) and Navigator (NAV) in the North Channel (Fig. 1). The TR area, located on the Tróia peninsula near the Sado Nature Reserve, is highly influenced by tidal hydrodynamics, leading to a shorter water residence time and reduced exposure to contamination from anthropogenic activities. On the other hand, GAM and NAV have lower hydrodynamics, allowing for the retention of fine-grained particles and the accumulation of contaminants and organic matter [35]. NAV is located near an industrial area and the port of Setúbal. The GAM area is located within the Sado Nature Reserve, close to oyster and aquaculture farms [36].

### Sampling Survey

A total of 18 sediment samples were collected from the intertidal zone, with three samples collected from each study area during two sampling campaigns: Aut20, corresponding to the autumn 2020 campaign conducted between November and December 2020, and Spr21, corresponding to the spring 2021 campaign conducted between May and June 2021. Samples were collected using a hand core, 20 cm deep, and 11 cm diameter, and were used for the physicochemical characterization of the sediment. Simultaneously, 42 cockles (*C. edule*) were collected in total, corresponding to seven individuals per study area and sampling campaign. Specimens were collected by hand at low tide, transported to the laboratory in sterile refrigerated boxes, and preserved in 70% ethanol at 4ºC until further processing.

### Sediment Characterization

#### Determination of Organic Matter, Organic Carbon, CaCO_3_, Grain Size and Environmental Parameters

The physicochemical characterization of the sediment followed methods previously described by [37], as the same sediment samples were used in the present study. Total organic matter (OM) and inorganic carbon (C_inorg_) fractions were determined following high-temperature combustion using a Leco Truspec micro-analyzer CHNS [38]. To isolate C_inorg_, sediment subsamples were combusted in a muffle furnace at 400 °C for 3 h to completely remove organic matter. Three replicate subsamples (2 mg each) of dried, homogenized sediment were measured before and after combustion, with a precision of 0.03% (dry weight). The organic carbon content (C_org_) was determined by subtracting the inorganic carbon fraction from total carbon.

The calcium carbonate (CaCO_3_) content was estimated by multiplying the inorganic carbon percentage by 8.332, assuming the predominant presence of calcite or aragonite. Total organic matter (TOM) content was determined by multiplying the organic carbon content (C_org_) by an adapated ‘Van Bemmelen factor’ of 2.5 for marine sediments [39, 40], which assumes that C_org_ constitutes 40% of the organic matter [41, 42]. Granulometric analysis was conducted to classify sediment fractions into gravel (> 2 mm), sand (2–0.063 mm), and fine fraction (< 0.063 mm) using hydraulic sieving after hydrogen peroxide (H_2_O_2_) treatment and disaggregation with pyrophosphate. Additionally, environmental parameters were recorded in situ directly from the sediment–water interface at each sampling site using a VWR pHenomenal® MU 600H multi-parameter probe. Temperature (°C), pH, salinity, and dissolved oxygen (mg/L), were obtained providing insights into the physico-chemical conditions at the time of collection.

#### Determination of Trace Metals

Trace metals quantification followed the protocol described in [37], ensuring consistency in data analysis across studies. Mercury (Hg) concentration was determined using thermal pyrolysis atomic absorption analysis (LECO 254 Advanced Mercury Analyser, AMA). The method involved pyrolyzing 15 mg of freeze-dried and ground sediment fraction lower than 2 mm at 750 °C in an oxygen atmosphere, capturing the sample on a gold amalgamator, and detecting it via atomic absorption spectrometry (AAS) using a silicon UV diode detector. Quality control was ensured using the MESS-4 certified reference material (CRM) (National Research Council of Canada), procedural blanks and duplicate samples. Hg concentrations were expressed in mg kg^−1^ of dry weight.

Other trace metals, namely arsenic (As), lithium (Li), manganese (Mn), nickel (Ni), chromium (Cr), uranium (U), barium (Ba), cobalt (Co), copper (Cu), zinc (Zn), lead (Pb), and molybdenum (Mo), were analyzed using Inductively Coupled Plasma—Mass Spectrometry (Perkin Elmer NEXIon 2000 C) after total digestion in a closed Teflon vessel microwave system (CEM MARS 5). Approximately 100 mg of homogenized freeze-dried and ground sediment (< 2 mm fraction) was digested with a mixture of Aqua Regia (36% hydrochloric acid and 60% nitric acid (HNO_3_); 3:1 v/v) and hydrofluoric acid (HF). The digestion process consisted of two steps: (1) heating to 180ºC for 25 min, followed by a 20-min hold; (2) neutralization of HF with 10 mL of a 4% boric acid solution at 170 °C for 10 min. After cooling, the solutions were transferred to 50 mL bottles and diluted with Type 1 ultrapure water. Before analysis, samples were diluted with HNO_3_ (2%). Trace metal recovery ranged from 85–115%, ensuring analytical accuracy.

Enrichment factors (EFs), calculated as previously described in [37], were used to assess trace metal enrichment relative to reference values. EF values close to 1 indicate minimal enrichment, whereas values above 1.5 suggest possible anthropogenic input.

### Sample Preparation, DNA Extraction, Amplification and Sequencing

The complete digestive tracts of 42 *C. edule* specimens, comprising seven individuals from each study area and seasonal campaign, were dissected and stored at −80 °C until processing. Before DNA extraction and sequencing, the seven digestive tracts from each group were pooled, resulting in six composite samples for DNA-based analysis. This pooling strategy was adopted because the aim of the study was to characterize an integrated gut microbiome profile representative of each site and seasonal campaign, rather than to assess inter-individual variability. Pooling seven individuals per group reduced the influence of individual-specific variation and provided a composite profile of the local cockle population under each environmental condition.

Total genomic DNA from cockles was extracted from 250 mg of each composite sample using the DNeasy PowerLyzer PowerSoil Kit (Qiagen), following the manufacturer´s protocol DNA concentration and the quality were assessed using a Qubit 4.0 Fluorometer (Invitrogen). The extracted DNA was sequenced using next-generation sequencing (NGS) to characterize the bacterial and fungal communities present in the cockle gut. The bacterial community was analysed by targeting the V3–V4 region of the 16S rRNA gene using primers 341 F 5′-CCTAYGGGRBGCASCAG-3′ and 806R 5′-GGACTACNNGGGTATCTAAT-3′) [43]. The fungal community was analysed by targeting the internal transcribed spacer regions 1(ITS1) region using the primers ITS5-1737F 5′-GGAAGTAAAAGTCGTAACAAGG-3′ and ITS2-2043R 5′-GCTGCGTTCTTCATCGATGC-3′) [44]. Library preparation and sequencing were performed by Novogene using an Illumina MiSeq platform with 250-bp paired-end reads. Raw reads were deposited into the NCBI Sequence Read Archive (SRA) database under accession number PRJNA1216054.

### Metabarcoding Data Analysis

Raw paired-end reads from the 16S rRNA and ITS datasets were processed separately using QIIME 2 (version 2024.10). Sequence quality filtering, denoising, dereplication, paired-end merging, and chimera removal were performed using the DADA2 denoise-paired plugin. Since the reads provided by Novogene had already undergone adapter removal and initial quality preprocessing, no additional fixed 5′ trimming or read truncation was applied in QIIME 2 (trim-left-f = 0, trim-left-r = 0, trunc-len-f = 0, and trunc-len-r = 0). All remaining parameters were kept at their default settings. This workflow yielded 586,414 non-chimeric 16S rRNA sequences and 418,006 non-chimeric ITS sequences.

Taxonomic assignment of bacterial and archaeal 16S rRNA gene ASVs was performed using the SILVA 138.1 reference database [34], whereas fungal ITS ASVs were classified using the UNITE database [35]. Taxonomic barplots were generated without removing unassigned taxa. ASVs lacking assignment at phylum level were retained as “Unassigned”. At genus level, ASVs without a confident genus-level classification were retained using the lowest available taxonomic information and labelled as unresolved categories, such as “Phylum_unassigned”, “uncultured”, or “Unknown_Family”. Relative abundances were calculated using the total retained ASV abundance, without renormalizing after removal of unassigned taxa. Alpha-diversity metrics, including Chao1 richness and Shannon and Simpson diversity indices, were calculated in QIIME 2 to quantify within-sample microbial diversity. Shared and unique bacterial ASVs among sampling sites were identified using presence/absence data derived from the ASV table, and Venn diagrams were generated using the Venny 2.1 web tool [45].

The Functional Annotation of Prokaryotic Taxa (FAPROTAX) database [46] was used to predict the functional profiles of the prokaryotic communities based on their taxonomic assignments, with particular emphasis on functions related to major biogeochemical cycles. As FAPROTAX infers functions from taxonomy, the results were interpreted as predicted functional potential rather than direct evidence of metabolic activity.

### Statistical Analysis

Principal Coordinates Analysis (PCoA) based on Bray–Curtis dissimilarities was used to explore differences in microbial community composition among samples at both the phylum and genus levels. PCoA was selected because Bray–Curtis dissimilarity is more appropriate for ecological community abundance data. Separate ordinations were performed for bacterial 16S rRNA gene and fungal ITS datasets at both taxonomic levels. Selected taxon vectors were added to the ordination plots to aid interpretation of the main taxa associated with sample separation. The PCoA analyses were performed using OriginPro V.2024b (OriginLab Corporation, Northampton, MA, USA), and the final figures were generated in R software [47] using the ggplot2 package [48].

Distance-based redundancy analysis (dbRDA; Legendre and Anderson, 1999) based on Bray–Curtis dissimilarities (Bray and Curtis, 1957) was used to explore the relationships between microbial community composition and sediment characteristics. Bacterial 16S rRNA gene and fungal ITS relative-abundance matrices were analyzed separately as response matrices, whereas sediment physicochemical variables were incorporated as explanatory variables. Prior to dbRDA, environmental variables were standardized to zero mean and unit variance to account for differences in units and scales. The analyses were performed in R using the vegan package. Environmental vectors were projected onto the ordination to indicate their direction of association with community composition. Given the limited number of composite samples, the dbRDA was interpreted descriptively.

## Results

### Sediment Characterization

Sediment characterization revealed distinct physicochemical profiles across the three study sites (Appendix A—Table A.1). The TR site was characterized by predominantly sandy (> 90%) with high CaCO_3_ content, low organic matter (OM), low organic carbon (C_org_), and lower trace metal concentrations, reflecting a limited contaminant retention capacity. In contrast, the NAV site exhibited the finest-grained sediments with higher OM and C_org_ values. This site also accumulated the highest concentrations of Zn, Cu, Pb, and Ni concentrations. Mercury (Hg) levels were notably elevated at NAV, with several samples showing enrichment factors (EF > 1.5) indicative of anthropogenic input, likely linked to industrial activities in the vicinity [37]. The GAM site presented a more heterogeneous grain-size distribution, including higher gravel content, and variable trace metal concentrations, particularly Cr and Ni. Regarding the in situ environmental parameters, dissolved oxygen (DO) concentrations were lowest at NAV during both sampling campaigns, particularly in spring. Conversely, during the spring campaign, TR showed the lowest temperature and the highest pH values, while GAM presented the highest overall DO concentrations.

### Gut Microbiome Diversity and Composition

**Fig. 2:**
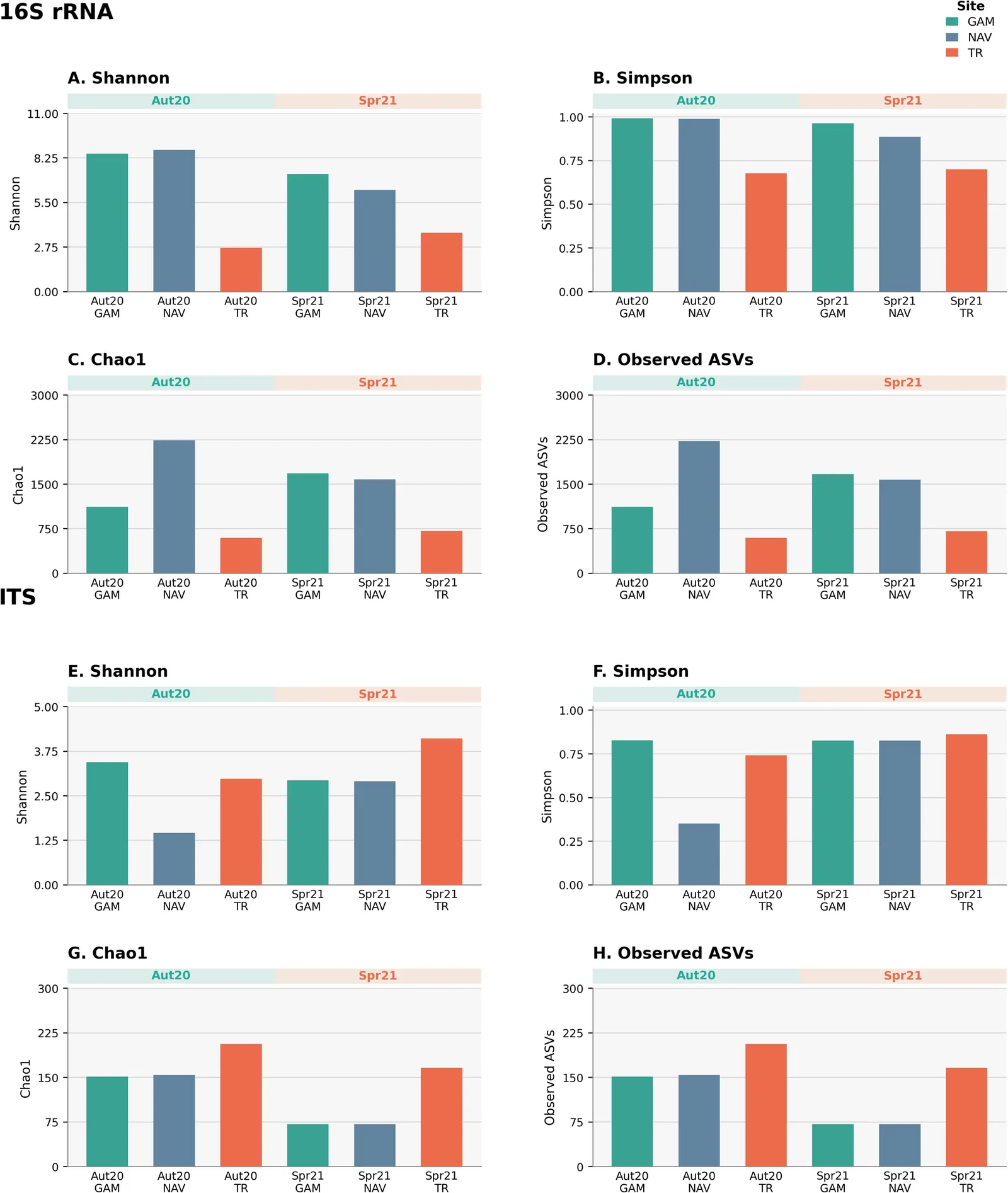
Alpha diversity metrics for the bacterial 16S rRNA gene and fungal ITS datasets. Shannon and Simpson diversity indices, Chao1 richness estimates, and observed ASVs are shown for each sampling site and season. Colours indicate sampling site: GAM, NAV and TR

A total of 586,414 high-quality non-chimeric 16S rRNA gene sequences were obtained from the intestinal tracts of *C. edule* collected from the three areas during the two seasons studied (Aut20GAM, Spr21GAM, Aut20NAV, Spr21NAV, Aut20TR and Spr21TR). Alpha diversity indices, including Shannon and Simpson, showed clear differences among areas and seasons (Fig. 2; Appendix A Table A.3). In autumn (Aut20), the highest bacterial diversity was observed in NAV (Shannon index: 8.74; Simpson index: 0.99), while the lowest was found in TR (2.71 and 0.68). During spring (Spr21), bacterial diversity decreased in GAM and NAV (Shannon index: 7.26 and 6.28, respectively) but increased in TR (3.63), although GAM and NAV gut samples still showed higher diversity than TR.

Samples collected in autumn from NAV and GAM showed greater bacterial diversity than those collected in spring, whereas TR samples were less diverse in autumn than in spring, as reflected by the Shannon and Simpson indices. The Chao1 estimator indicated the highest richness in the NAV sample in autumn (Chao1 ≈ 2,235) and in the GAM sample in spring (Chao1 ≈ 1,680), suggesting that these samples contained a greater number of taxa. In contrast, the TR samples exhibited lower richness in both seasons (Chao1 ≈ 595 in autumn and ≈ 712 in spring), supporting the pattern observed for the Shannon and Simpson indices.

A total of 418.006 high-quality non-chimeric ITS reads were obtained from the intestinal samples, corresponding to 71–206 observed ITS ASVs per pooled sample (Aut20GAM, Spr21GAM, Aut20NAV, Spr21NAV, Aut20TR and Spr21TR). The richness and evenness of the fungal communities were estimated using Chao1, Shannon and Simpson indices (Fig. 2). The most diverse samples were Aut20GAM and Spr21TR, with Shannon and Simpson indices of 3.44 and 0.83, and 4.10 and 0.86, respectively. In contrast, NAV samples showed lower diversity, particularly in autumn (Aut20NAV, Shannon index: 1.45; Simpson index: 0.35). Chao1 richness was highest in TR (Aut20TR: 206; Spr21TR: 166), whereas spring GAM and NAV samples showed the lowest richness (Chao1: 71 in both cases).

**Fig. 3:**
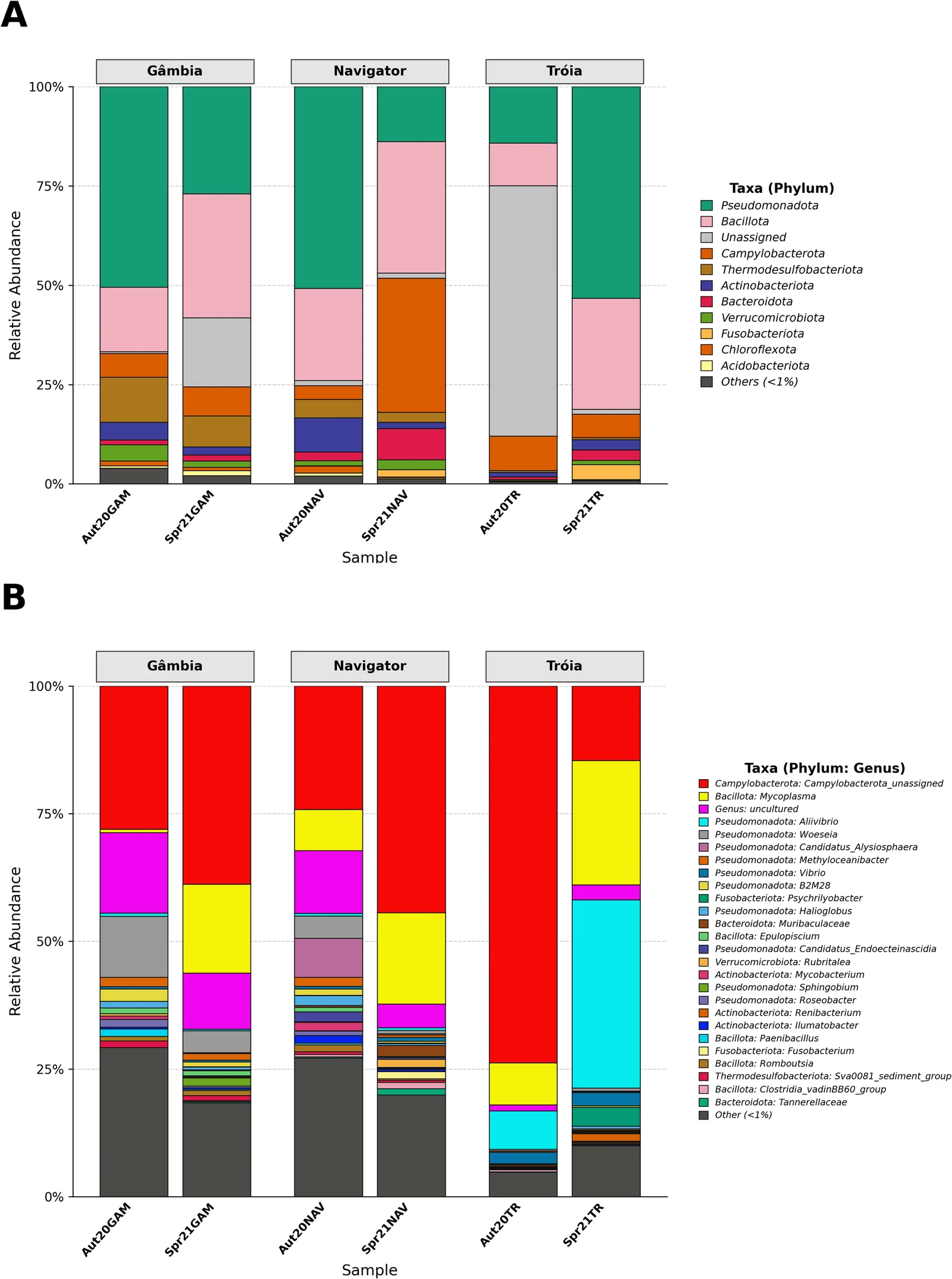
Relative abundances of prokaryotic (16S rRNA gene) phyla (**A**) and genera (**B**) in the gut microbiome of *Cerastoderma edule* from three different areas (NAV (Navigator), GAM (Gâmbia) and TR (Tróia)) across two different seasons (Aut20 and Spr21)

The main bacterial phyla represented in the intestinal tracts of the cockles studied were *Pseudomonadota*, *Bacillota*, and *Campylobacterota* across all sites and seasons (Fig. 3A). A notable fraction of sequences remained unassigned at the phylum level, particularly in the Aut20TR sample (~ 63%), suggesting the presence of poorly characterized lineages. *Pseudomonadota* and *Bacillota* dominated the communities in GAM and NAV, whereas TR showed a marked seasonal contrast, shifting from an autumn community with a high proportion of unassigned and *Pseudomonadota*, *Bacillota*, *Campylobacterota* sequences to a spring community dominated by *Pseudomonadota* (53.29%) and *Bacillota* (27.96%). Within *Bacillota*, the genus *Mycoplasma* was detected in all samples and reached its highest relative abundance in the gut of *C. edule* from TR during spring.

The phylum *Pseudomonadota* was particularly abundant in the GAM and NAV samples in autumn, with relative abundances of 50.43% and 50.78%, respectively. In spring, it reached its highest relative abundance in the TR sample (53.29%; Fig. 3A). The most abundant members of this phylum were *Woeseia* and *Vibrio*. *Woeseia* was especially abundant in the GAM and NAV samples in autumn, particularly in Aut20GAM (24.59%), whereas its abundance in TR remained below 1% in both seasons.

Other genera associated with marine environments include *Halioglobus*, mainly found in the NAV and GAM samples, indicating that these nitrogen-reducing bacteria can thrive in metal-enriched sediments. The predominance of the phylum *Bacillota* was observed at the GAM and NAV sites during both seasons, with relative abundances of 16.28–23.18% in autumn and 31.17–33.14% in spring, while its abundance in TR was lower in autumn (10.75%) but increased in spring (27.96%). The phylum *Campylobacterota* showed a similar relative abundance in GAM in both seasons (autumn: 5.99%; spring: 7.29%). However, a pronounced increase in relative abundance was observed in the spring NAV sample (33.79%), whereas TR displayed more moderate values (8.69% in autumn and 5.92% in spring). The phyla *Actinobacteriota* and *Bacteroidota* were especially represented in NAV samples, particularly in autumn, while *Verrucomicrobiota* and *Thermodesulfobacteriota* were particularly abundant in the gut of *C. edule* from GAM. Other, less abundant phyla, including *Fusobacteriota* and *Acidobacteriota*, were more conspicuous in spring.

**Fig. 4:**
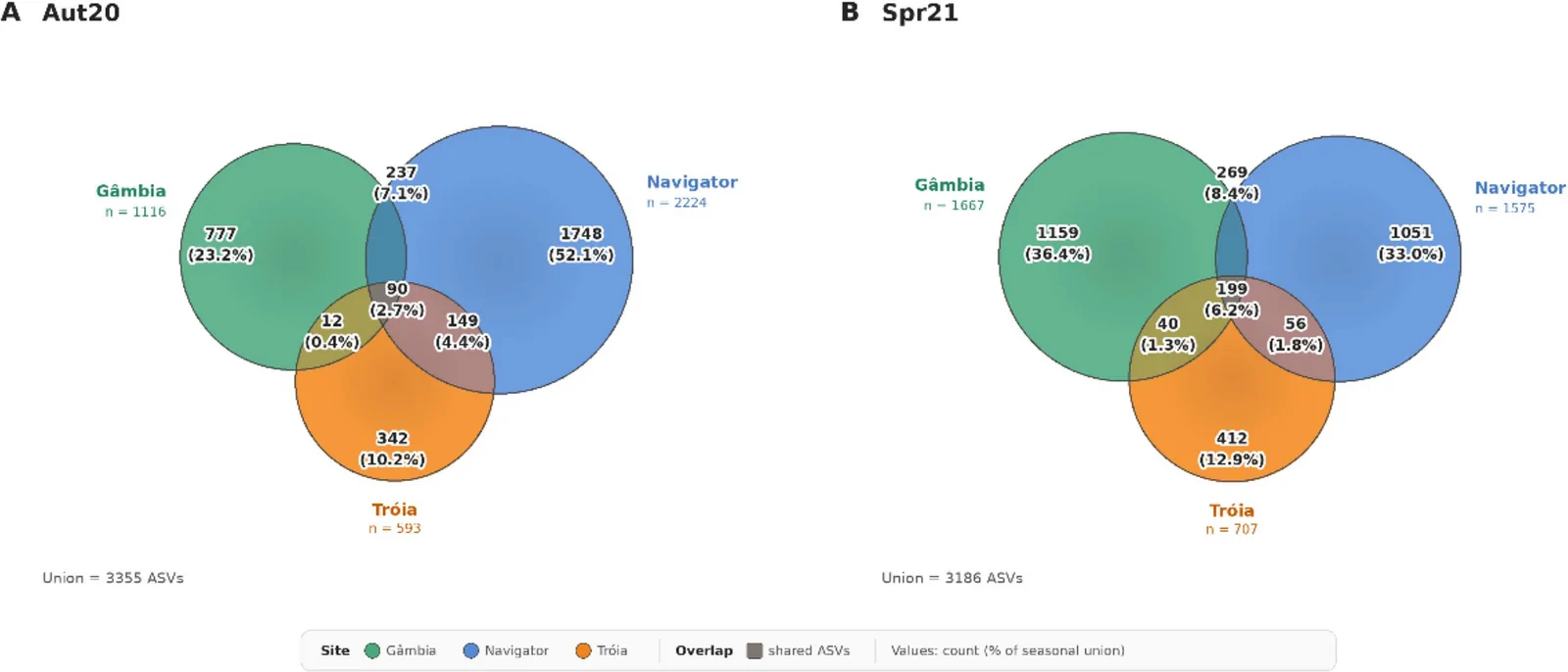
Venn diagrams showing shared and unique ASVs among sampling sites in Aut20 and Spr21. Numbers indicate ASV counts, with percentages in parentheses representing the proportion relative to the seasonal ASV union. Colours indicate the sampling sites: Gâmbia, Navigator and Tróia. Blended colours highlight overlapping regions corresponding to shared ASVs among sites. Circle areas are scaled according to the total number of ASVs detected at each site

The Venn diagrams for the bacterial communities revealed clear differences in the distribution of genera between sites and seasons (Fig. 4). The number of genera shared by all three sites increased from 90 in autumn (Aut20GAM, Aut20NAV and Aut20TR) to 199 in spring (Spr21GAM, Spr21NAV and Spr21TR), corresponding to 2.7% and 6.2% of the seasonal ASV union. In autumn, NAV harboured the highest number of unique ASVs (1748; 52.1%) followed by GAM (777; 23.2%) and TR (342; 10.2%). In spring, GAM and NAV still contained large pools of site-specific ASVs (1159; 36.4% and 1051; 33.0%, respectively), whereas TR showed fewer unique ASVs (412; 12.9%). This pattern indicates that GAM and NAV supported more site-specific bacterial assemblages, while TR hosted a comparatively smaller unique ASV fraction. The number of ASVs exclusively shared between GAM and TR was low in both seasons (12 in Aut20 and 40 in Spr21), highlighting marked compositional differences between these two sites.

**Fig. 5:**
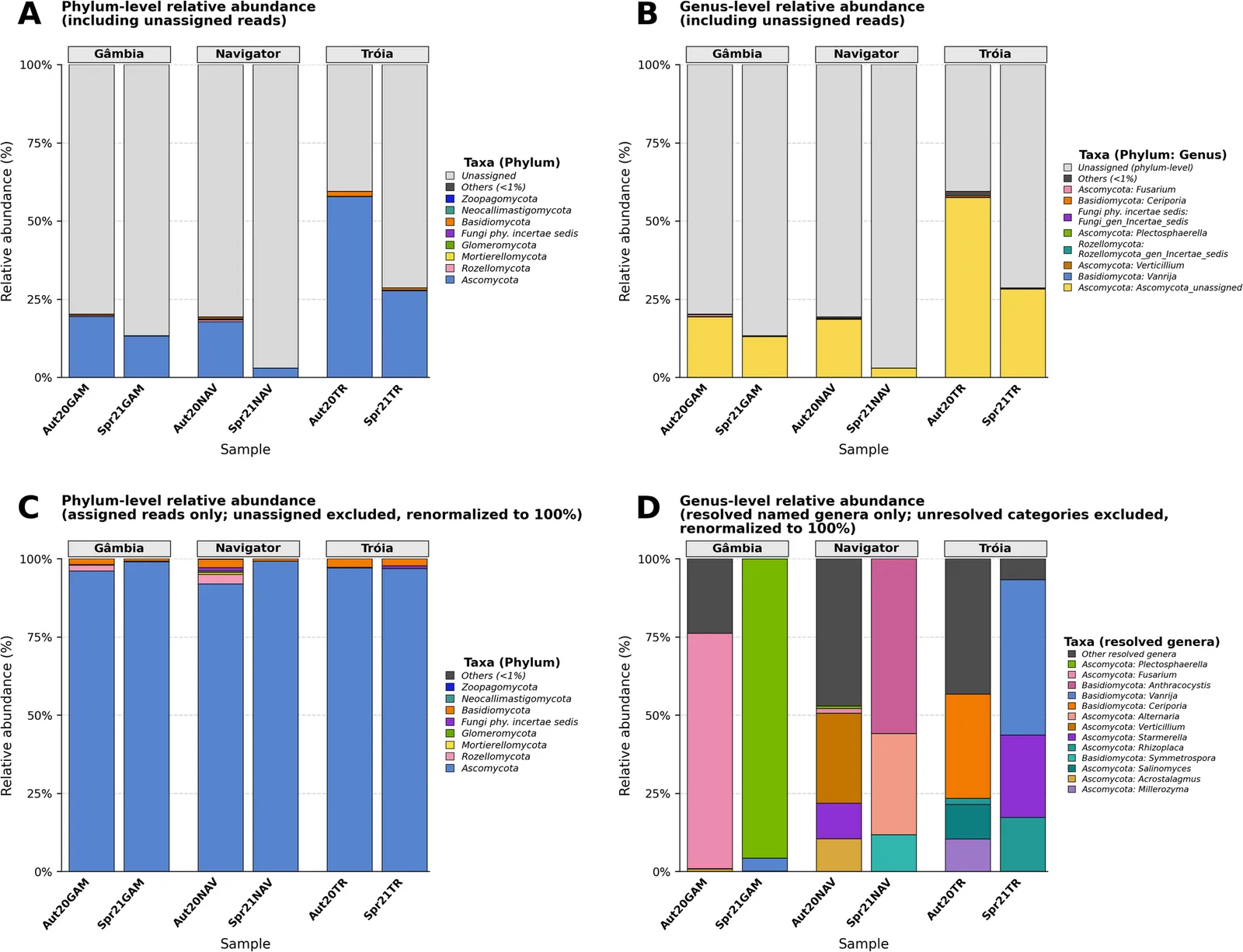
Relative abundances of fungal (ITS) phyla (A, C) and genera (B, D) in the gut microbiome of *Cerastoderma edule* from three different areas (NAV (Navigator), GAM (Gâmbia) and TR (Tróia)) during two different seasons (Aut20 and Spr21). Panels A and B show the relative abundances including unassigned sequences, whereas panels C and D show the relative abundances recalculated after excluding sequences classified as unassigned

The fungal communities present in the gastrointestinal tracts of *C. edule* from the three sites and seasons exhibited similar diversity indices but marked differences in relative abundance patterns among the taxonomically assigned fraction (Fig. 5). However, most ITS reads (approximately 70–97%) could not be confidently assigned to higher taxonomic ranks, limiting the ecological interpretation of fungal community composition. Within the fraction that could be classified, *Ascomycota* was the dominant phylum at all sites, followed by much lower contributions of *Basidiomycota* and, in autumn, minor proportions of *Fungi incertae sedis, Mortierellomycota, Glomeromycota, Rozellomycota* and *Zoopagomycota*.

*Ascomycota* reached its highest relative abundance in TR, particularly in autumn (57.8%) and, to a lesser extent, in spring (27.7%), whereas GAM and NAV showed lower proportions of this phylum and a higher fraction of unassigned reads. At the genus level, most ascomycetous sequences remain unresolved (*Ascomycota*_unassigned). Among the identified genera, *Fusarium* was conspicuous in Aut20GAM and, to a lesser extent, in Aut20NAV, while TR samples harboured a mixture of filamentous saprotrophs, including *Plectosphaerella Ceriporia,* and *Verticillium*. *Basidiomycota*, although present at low relative abundances (< 2% of total reads), also displayed site- and season-specific patterns. Autumn samples from TR and NAV contained a diverse set of basidiomycetous taxa, including the wood-decaying genera *Ceriporia*, whereas the autumn GAM sample was characterized by the occurrence of *Fusarium* a saprotrophic sordariomycete. *Basidiomycetous* yeasts such as *Vanrija* were sporadically detected, mainly in TR. *Rozellomycota* was detected at very low relative abundance and mainly in the autumn samples from GAM and NAV.

**Fig. 6:**
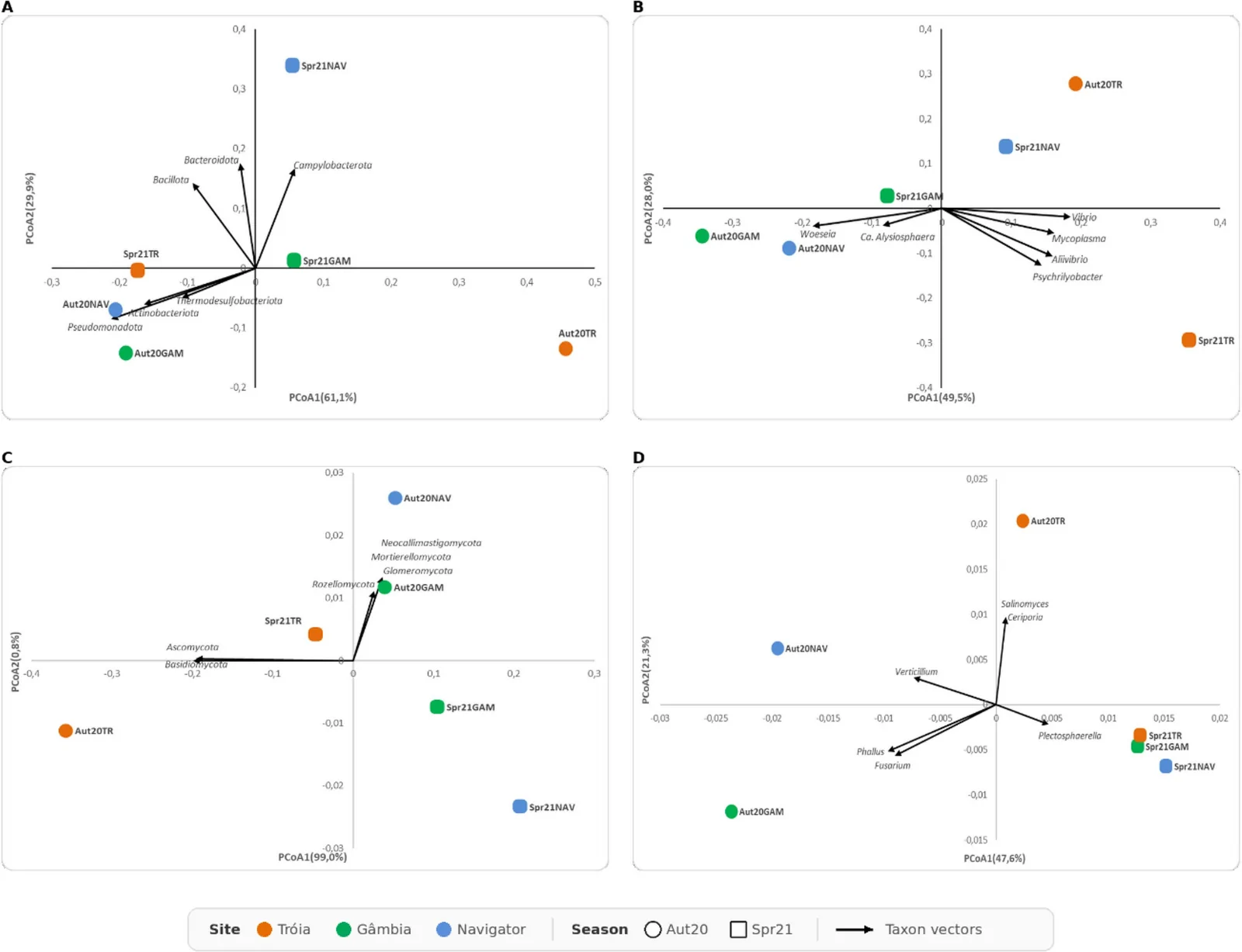
Principal Coordinates Analysis (PCoA) based on Bray–Curtis dissimilarities of the *C. edule* gut microbiome at the (**A**) bacterial phylum, (**B**) bacterial genus, (**C**) fungal phylum and (**D**) fungal genus taxonomic levels. Colours indicate sampling site: Tróia (TR), Gâmbia (GAM) and Navigator (NAV). Symbols indicate season: circles, Aut20; squares, Spr21. Arrows indicate selected taxon vectors associated with the ordination axes

Principal Coordinates Analysis (PCoA) based on Bray–Curtis dissimilarities was performed at phylum and genus levels to explore differences in gut microbial community composition among samples (Fig. 6). At the bacterial phylum level (Fig. 6A), the first two PCoA axes explained 61.1% and 29.9% of the variation, respectively. TR samples were separated from GAM and NAV samples mainly along PCoA1, whereas Spr21NAV was clearly separated along PCoA2. Taxon vectors indicated that bacterial phylum-level differences were mainly associated with *Pseudomonadota, Bacillota, Bacteroidota and Campylobacterota*.

At the bacterial genus level (Fig. 6B), PCoA1 and PCoA2 explained 49.5% and 28.0% of the variation, respectively. TR samples were separated from the remaining sites, particularly along PCoA1, while GAM and NAV samples occupied closer positions in the ordination space. The main taxon vectors associated with this separation included *Vibrio, Mycoplasma, Aliivibrio, Psychrilyobacter, Woeseia and Candidatus Alysiosphaera.*

Fungal communities displayed a different ordination pattern. At the ITS phylum level (Fig. 6C), PCoA1 explained most of the variation (99.0%), while PCoA2 explained 0.8%. Aut20TR was clearly separated from the remaining samples along PCoA1, whereas Aut20NAV and Spr21NAV occupied opposite regions along PCoA2. The main fungal phylum vectors were associated with *Ascomycota, Basidiomycota, Rozellomycota, Neocallimastigomycota, Mortierellomycota and Glomeromycota.*

At the ITS genus level (Fig. 6D), PCoA1 and PCoA2 explained 47.6% and 21.3% of the variation, respectively. Autumn samples showed greater dispersion across the ordination space, with Aut20TR and Aut20GAM occupying opposite regions, whereas spring samples were positioned closer to each other on the positive side of PCoA1. Taxon vectors indicated associations with *Salinomyces, Ceriporia, Verticillium, Plectosphaerella, Phallus and Fusarium.*

### Predict Bacterial Functional Profiles of the Gut Microbiome

**Fig. 7:**
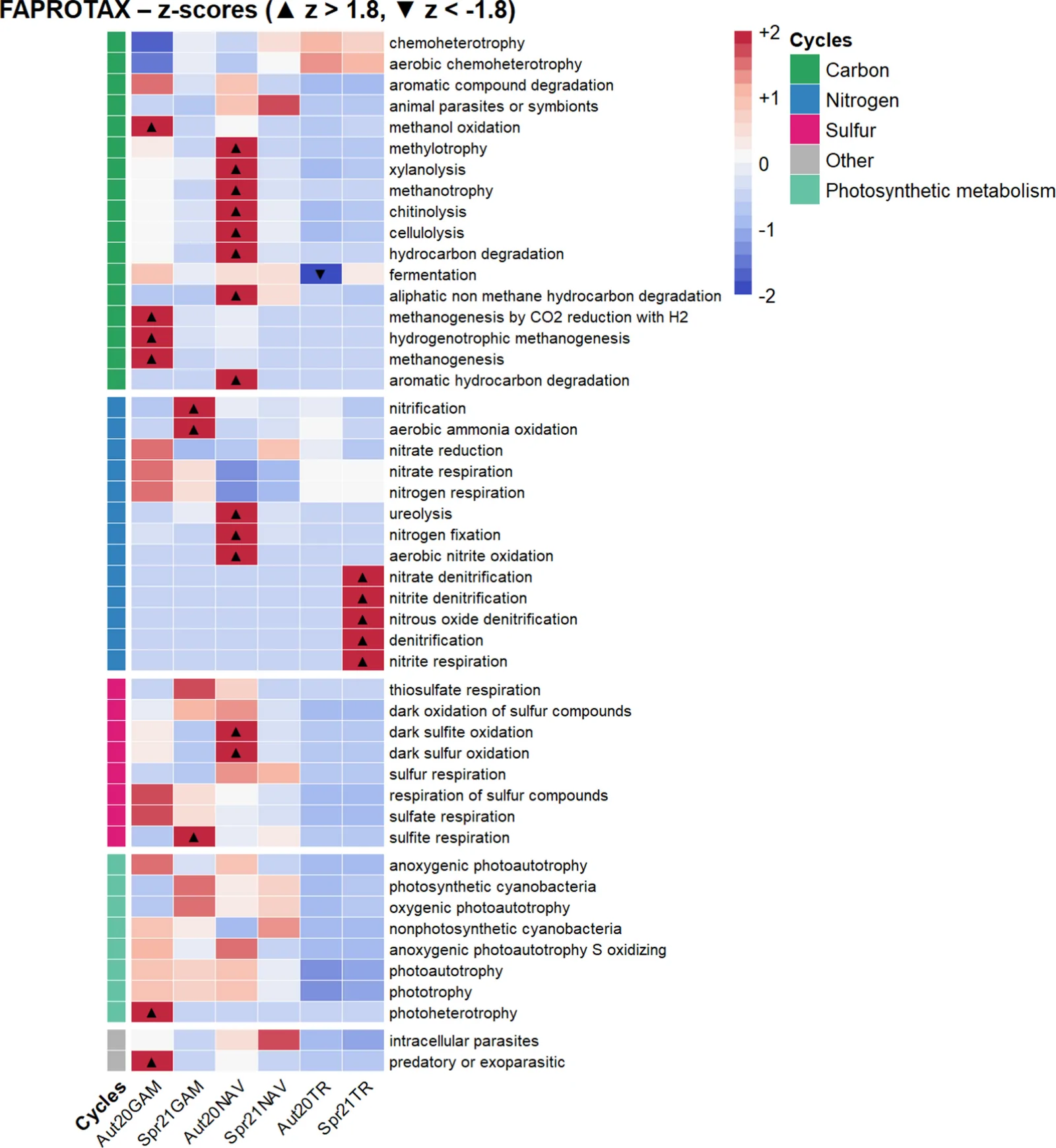
Predicted functional profiles of the *C. edule* gut microbiome from the Sado Estuary

The predicted functional profile of the C. *edule* gut microbiome, inferred with FAPROTAX, revealed metabolic activities mainly related to carbon, nitrogen and sulfur cycling (Fig. 7), with clear differences among sites (GAM, NAV and TR) and seasons (Aut20, Spr21). Putative chemoheterotrophy and aerobic chemoheterotrophy dominated all samples, indicating that the gut microbiota is largely sustained by the degradation and fermentation of organic substrates. Predicted functions associated with the breakdown of complex polymers (xylanolysis, cellulolysis and chitinolysis) and fermentation showed particularly high z-scores in autumn samples from GAM and NAV, suggesting an enhanced capacity for detrital organic-matter remineralization at these sites.

Potential sulfur cycling functions were especially prominent in GAM in both seasons and, to a lesser extent, in NAV, mainly through sulfate respiration and the broader “respiration of sulfur compounds” category. In contrast, TR samples displayed much lower contributions of sulfur-respiration pathways, consistent with the lower organic-matter and contaminant loads previously described for this site.

Nitrogen-related functions predicted using FAPROTAX also differed among sites. Nitrogen fixation was most enriched in the Aut20NAV sample, indicating a higher potential for biological N inputs in this industrially impacted area, whereas the other sites showed lower but detectable nitrogen-fixation signals. Nitrification (aerobic ammonia and nitrite oxidation) and nitrate reduction/respiration were comparatively more important in GAM, particularly in spring. In contrast, the full denitrification pathway (nitrate, nitrite and nitrous-oxide reduction) was mainly associated with the Spr21TR sample.

Carbon-degradation pathways displayed a marked site specificity. Aromatic-compound degradation was strongly enriched in Aut20GAM, while hydrocarbon-degradation functions peaked in Aut20NAV. Photosynthesis-related functions (oxygenic and anoxygenic photoautotrophy, photosynthetic cyanobacteria) were more prominent in GAM and NAV, particularly in spring, whereas TR samples showed comparatively lower contributions of photoautotrophic pathways and were dominated by heterotrophic and nitrogen-cycling functions.

### Correlation Between Sediment Properties and Cerastoderma Edule Gut Microbial Composition

**Fig. 8:**
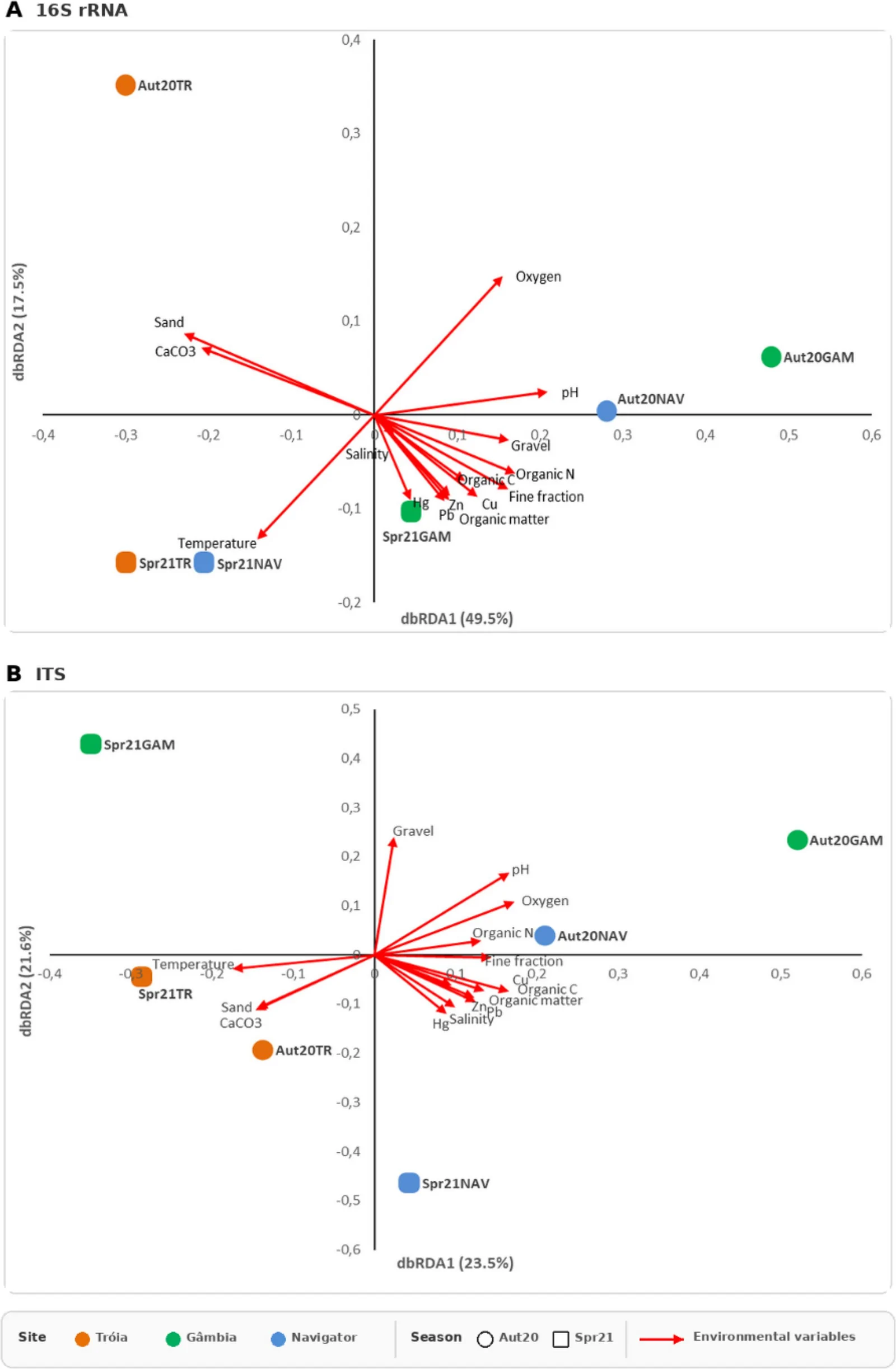
Distance-based redundancy analysis (dbRDA) based on Bray–Curtis dissimilarities showing the relationships between sediment characteristics and (**A**) bacterial 16S rRNA gene and (**B**) fungal ITS community composition. Colours represent the sampling sites (Gâmbia (GAM), Navigator (NAV) and Tróia (TR)), symbols represent seasons (Aut20 and Spr21), and arrows indicate the direction of association of environmental variables with microbial community composition

Distance-based redundancy analysis (dbRDA) revealed differences in bacterial and fungal gut community composition among sampling sites and seasons (Fig. 8). For bacterial communities, dbRDA1 and dbRDA2 explained 49.5% and 17.5% of the total variation, respectively (Fig. 8A). Samples from TR were positioned toward negative dbRDA1 scores, whereas the autumn samples from GAM and NAV occupied the positive region of this axis. Oxygen and pH were associated with positive dbRDA1 scores, while sand and CaCO₃ were oriented toward negative dbRDA1 scores. Fine sediment fraction, organic matter, organic carbon and nitrogen, and several trace metals were primarily associated with positive dbRDA1 and negative dbRDA2 scores.

Fungal communities displayed a different ordination pattern (Fig. 8B), with dbRDA1 and dbRDA2 accounting for 23.5% and 21.6% of the total variation, respectively. The autumn GAM sample was positioned toward positive values of both axes, whereas the spring GAM sample showed negative dbRDA1 and strongly positive dbRDA2 scores. NAV samples were mainly separated along dbRDA2, while TR samples occupied the negative region of dbRDA1. Gravel, pH and oxygen were associated with positive dbRDA2 scores, whereas temperature, sand and CaCO₃ were oriented toward negative dbRDA1 scores. Organic matter, fine sediment fraction and trace metals were mainly associated with positive dbRDA1 and negative dbRDA2 scores.

The ordination suggests that sediment characteristics and trace-metal gradients are associated with spatial and seasonal differences in gut microbial community composition. Given the limited number of composite samples, these patterns were interpreted descriptively, and no causal relationships were inferred.

## Discussion

### Sediment Characteristics Reflect Contrasting Environmental Conditions Across Sites

Sediment characterization revealed clear spatial differences among the three study areas, reflecting different hydrodynamic conditions, sediment composition, and anthropogenic pressures [35, 49]. The predominantly sandy matrix at TR characterized by high CaCO₃ content, low OM, low C_org_ carbon and lower trace metal concentrations, points to points to a high-energy hydrodynamic environment with a reduced capacity for contaminant retention. In contrast, the depositional nature of the NAV site is highlighted by finer sediments, elevated OM and C_org_ contents, which facilitate the accumulation of Zn, Cu, Pb, Ni and Hg concentrations. Meanwhile, the GAM site represents an intermediate, more heterogeneous environment, marked by higher gravel content and localized variations in chromium and nickel concentrations. These sediment profiles are consistent with previous assessments of the same study area, which identified stronger anthropogenic influence at NAV, including metal enrichment in several samples [37]. This environmental gradient provides a relevant framework for interpreting spatial variation in the gut microbiome of *C. edule*.

### Gut Microbiome Diversity and Composition Reflect Site-specific Sediment Conditions

The gut microbiome of *C. edule* showed clear spatial and seasonal variation, suggesting that local sediment characteristics and anthropogenic pressures may influence host-associated microbial communities [11, 13, 14, 50]. Bacterial diversity was generally higher in NAV and GAM than in TR, particularly during the autumn campaign, which may reflect the greater environmental heterogeneity and organic enrichment observed at the more impacted sites. Seasonal variation also appeared to reduce differences among sites during the spring campaign, suggesting that environmental drivers such as temperature and salinity may partially homogenize gut microbial communities. In contrast, the lower bacterial diversity observed at TR, together with its sandy sediments, lower organic matter content and lower trace metal concentrations, suggests a more distinct gut bacterial profile under comparatively less contaminated conditions.

The bacterial communities from TR were mainly characterized by higher relative abundances of *Mycoplasma* and *Vibrio*. *Mycoplasma* is commonly reported in bivalve microbiomes and may represent an important component of host-associated bacterial communities [5, 11, 50]. Its higher representation at TR may therefore be consistent with less disturbed sediment conditions, although this interpretation should be made cautiously because host physiology and other unmeasured factors may also influence its abundance [51, 52]. *Vibrio* was also more abundant at TR, particularly during the spring campaign. Although some *Vibrio* species are pathogenic, this genus is also a natural component of marine bivalve microbiomes and may contribute to nutrient acquisition, organic matter degradation and host-associated metabolic processes [53, 54]. Its higher representation at TR may also reflect adaptation to the comparatively lower organic matter availability at this site. Therefore, its higher abundance at TR should not be interpreted solely as an indicator of disease, but rather as part of the natural and environmentally responsive gut microbiome of *C. edule*.

In contrast, NAV and GAM showed higher relative abundances of *Pseudomonadota*, *Bacillota* and other metabolically versatile bacterial groups. These taxa include bacteria frequently associated with organic enrichment, sediment disturbance and contaminant exposure, which is consistent with the higher organic matter content and trace metal concentrations observed at these sites [4, 14, 26]. Among its members the genus *Woeseia* is commonly associated with deep-sea, coastal and continental shelf environments, where it has been reported to play an important role in the cycling of detrital proteins in marine benthic ecosystems. The increased contribution of *Campylobacterota* at NAV may also be related to finer sediments enriched in organic matter and to anthropogenic inputs. Genera within this phylum, such as *Arcobacter* and *Campylobacter*, have been associated with polluted aquatic environments and have been detected in edible bivalves [55–57]. Their occurrence in the gut microbiome of *C. edule* may therefore indicate exposure to environmentally derived bacteria potentially linked to organic enrichment or fecal contamination, reinforcing the role of filter-feeding bivalves as sentinels of estuarine environmental quality.

Some bacterial groups detected in NAV and GAM, including *Fusobacteriota, Acidobacteriota* and sulfur-associated or sediment-related taxa, may reflect the influence of sediment ingestion and local environmental conditions on the gut microbiome. *Fusobacteriota* has been associated with anaerobic or sediment-rich gut environments, whereas *Acidobacteriota* are more commonly reported in soils and sediments than in bivalve guts [9, 58]. Their presence in *C. edule* may therefore indicate transient environmental acquisition rather than stable host association. This interpretation is supported by the fact that sediment and interstitial water microbiomes were not analyzed in the present study, limiting our ability to distinguish resident gut bacteria from environmentally derived taxa.

The fungal component of the *C. edule* gut microbiome also exhibited variation among sites and seasonal campaigns. However, the interpretation of these data is limited by the high proportion of unassigned ITS sequences. The recurrent dominance of poorly resolved *Ascomycota*-affiliated lineages suggests that the cockle gut may harbour fungal assemblages associated with marine or estuarine organic matter, sediment-derived fungi and opportunistic colonizers [17, 18, 59]. These fungi are commonly associated with organic matter decomposition, nutrient cycling and microbial interactions in marine sediments, suggesting that the digestive tract may act as a transient habitat for environmentally derived fungal taxa. Nevertheless, the significant proportion of unassigned taxa limits the capacity to provide a robust assessment of whether these fungi constitute a true host-associated core mycobiome [17]. Consequently, patterns previously interpreted as indicative of fungal stability or resilience should be viewed with a degree of caution, as they may to some extent reflect current limitations in marine fungal reference databases rather than genuine ecological stability [17, 60, 61].

Low-abundance fungal groups, including *Rozellomycota* and other poorly resolved lineages, were mainly detected in the more anthropogenically influenced sites. *Rozellomycota* comprises intracellular parasitic and symbiotic fungi commonly found in aquatic environments and sediments and has been reported from metal-impacted habitats [62]. However, its ecological role in the gut of *C. edule* remains unclear [63]. The presence of these fungal lineages may reflect environmental acquisition from sediment or water, tolerance to contaminated conditions, or opportunistic colonization of the digestive tract. Future studies including sediment, water and host tissue compartments will be necessary to distinguish resident fungal taxa from transient environmental fungi and to clarify their functional relevance in bivalve gut ecosystems.

The PCoA analyses suggest that sampling sites exerted a stronger influence on gut microbial composition than season, particularly for bacterial communities. The clear separation of TR from NAV and GAM suggests that local environmental conditions, including differences in sediment characteristics and anthropogenic pressures, play a major role in shaping the bacterial assemblages associated with *C. edule.* In contrast, the closer clustering of NAV and GAM is consistent with their shared exposure to higher organic matter content and trace-metal contamination, which may favour metabolically versatile taxa such as members of the phylum *Pseudomonadota*.

Fungal communities displayed a different ordination pattern. At the phylum level, the first PCoA axis explained almost all of the observed variation (99%), indicating that fungal communities were dominated by a single major compositional gradient. At the genus level, autumn samples showed greater dispersion than spring samples, suggesting higher temporal variability in fungal community composition rather than a purely site-driven pattern. This greater variability may reflect seasonal changes acting in combination with local environmental conditions. Moreover, the high proportion of unclassified ITS sequences, together with the dominance of *Ascomycota*, likely reduced the taxonomic resolution of the fungal ordination and highlights the current limitations of fungal reference databases for environmental microbiome studies.

These findings support the hypothesis that bacterial communities are primarily shaped by local environmental conditions, whereas fungal communities appear to respond to a combination of environmental and seasonal factors.

### Predict Bacterial Functional Profiles Indicate Potential Responses to Sediment Conditions

Predicted bacterial functional profiles indicated site-specific differences in the putative metabolic potential of the *C. edule* gut microbiome. Functions associated with sulfur respiration were more represented in NAV and GAM, particularly in samples from the more anthropogenically influenced areas. These functions are commonly associated with sulfur-transforming bacteria involved in estuarine sediment metabolism, organic matter degradation and redox processes [9, 64]. The lower contributions of sulfur-respiration pathways in TR samples are consistent with the lower organic-matter and contaminant loads previously described for this site.

Nitrogen-related functions also varied among sites and seasonal campaigns. The higher predicted nitrogen fixation potential in Aut20NAV may indicate a greater contribution of bacterial groups involved in biological nitrogen inputs in this industrially influenced area. In GAM, particularly during spring, the stronger signals of nitrification and nitrate respiration suggest potential nitrogen turnover in the aquaculture-influenced sector. By contrast, the enrichment of denitrification-related functions in Spr21TR may reflect conditions favoring natural nitrogen removal processes in this less impacted area [10, 65, 66].

Functions related to aromatic compound and hydrocarbon degradation were predicted mainly in Aut20GAM and Aut20NAV, respectively. This pattern is consistent with the higher anthropogenic influence at these sites and with the presence of bacterial groups known for metabolic versatility and degradation of organic pollutants, including taxa related to *Pseudomonas* and *Alcanivorax* [67–69]. These predicted functions may indicate that bacterial communities associated with the gut of *C. edule* reflect exposure to sedimentary organic enrichment and contamination-related pressures [23].

Nevertheless, these results should be interpreted cautiously because FAPROTAX infers potential functions from taxonomic assignments rather than directly measuring functional genes, gene expression or metabolic activity [46, 66]. The simultaneous prediction of apparently contrasting processes, such as aerobic and anaerobic pathways, sulfur oxidation and sulfur reduction, or methanogenesis and aerobic carbon oxidation, may reflect the coexistence of distinct microbial niches, the presence of facultative or metabolically versatile taxa, mixed environmental sources associated with sediment ingestion, or limitations of taxonomy-based functional inference. Therefore, FAPROTAX-derived profiles should be considered hypothesis-generating indicators of potential functional capacity rather than direct evidence of active biogeochemical processes. Future metagenomic, metatranscriptomic or metabolomic analyses will be necessary to validate the actual metabolic activity of these microbial communities.

### Sediment Properties and Trace Metal Gradients are Associated With *C. edule gut* Microbial Composition

The association between sediment properties and gut microbial composition suggests that local environmental conditions contribute to structuring the *C. edule* microbiome. At NAV, finer sediments, higher organic matter content and increased concentrations of trace metals, including Zn, Cu, Pb, Ni and Hg, were associated with bacterial groups commonly linked to organic enrichment, sediment influence and disturbed environments. In particular, the higher representation of *Campylobacterota* at NAV may reflect exposure to fine, organic-rich sediments and anthropogenic inputs, potentially linked to nearby industrial activities [4, 26, 35].

GAM also showed evidence of anthropogenic influence, although with a more heterogeneous sediment profile. The variable concentrations of Cr and Ni, together with the influence of aquaculture activities, may help explain the occurrence of metabolically versatile bacterial taxa and predicted functions related to organic compound degradation. These patterns suggest that both industrial and aquaculture-related pressures may contribute to differences in gut microbial composition between NAV, GAM and TR.

In contrast, TR was characterized by sandy sediments, lower organic matter content, and lower trace metal concentrations. The gut microbiome at this site was more strongly associated with taxa such as *Mycoplasma* and *Vibrio* and showed a lower contribution of bacterial groups typically linked to organic enrichment or contaminant-associated conditions. This pattern supports the interpretation of TR as a comparatively less impacted site, although the absence of sediment and interstitial water microbiome data prevents direct confirmation of whether these microbial taxa originated from the surrounding environment or from host-associated processes.

The fungal results also suggest a possible relationship with sediment conditions, but these patterns should be interpreted more cautiously. The association between low-abundance fungal lineages, including *Rozellomycota*, and the more anthropogenically influenced sites may reflect environmental acquisition, tolerance to disturbed conditions or opportunistic colonization. However, because many ITS sequences remained unassigned, and because sediment fungal communities were not analyzed, it is not possible to determine whether these taxa represent resident gut fungi or transient sediment-derived fungi [17, 60, 61].

Overall, the observed bacterial and fungal patterns suggest that *C. edule* gut microbial communities reflect a combination of sediment properties, trace metal gradients, anthropogenic activities, and seasonal variability. Bacterial communities appeared more directly associated with organic enrichment and contamination-related gradients, whereas fungal communities provided complementary but more taxonomically uncertain information. These findings support the potential use of bivalve-associated microbiomes as complementary tools for estuarine environmental assessment, while also highlighting the need to integrate host condition, tissue metal concentrations, and sediment and water microbiomes in future studies.

## Conclusions

This study highlights the potential of the *Cerastoderma edule* gut microbiome as a complementary biomonitoring tool for assessing estuarine environmental conditions. The observed variation in bacterial and fungal communities across sites and seasonal campaigns suggests that gut-associated microbiomes can reflect the interplay between natural environmental gradients and localized anthropogenic pressures. However, these structural patterns must be interpreted cautiously, as confounding host-level factors, such as sex, physiological condition, health status and population density, were not evaluated in this study.

Our findings show that characterizing microbial functional traits provides valuable ecological context, emphasizing that shifts in taxonomic diversity do not automatically equate to a balanced or functionally stable ecosystem. Nevertheless, the functional profiles inferred in this study should be considered as indicators of potential metabolic capacity rather than direct evidence of active microbial processes. To validate these microbial activities in situ and clarify the exact contributions of specific taxa to host physiology and estuarine biogeochemical cycling, subsequent research should employ multi-omic methodologies, including shotgun metagenomics, metatranscriptomics, or metabolomics.

Finally, subsequent monitoring programs should integrate sediment and interstitial water microbiomes, cockle tissues bioaccumulation profiles, host biological traits, and a broader spectrum of chemical stressors, such as organic and emerging contaminants. Extending this approach to other estuarine macroinvertebrates and to geographically distinct *C. edule* populations would strengthen their applicability for biodiversity conservation, environmental risk assessment, and sustainable estuarine management under increasing anthropogenic pressure and climate change.

## Supplementary Information

Below is the link to the electronic supplementary material.

Supplementary file1 (DOCX 33 KB)

## Data Availability

The datasets generated during the current study are available in the NCBI Sequence Read Archive (SRA) under accession number PRJNA1216054.
