# Supplementary material for: Bivalve Gut Microbiome Responses to Sediment Characteristics and Anthropogenic Activities in Cerastoderma edule (Linnaeus, 1758)
Source: Microb Ecol. 2026 Aug 15;89:207. doi: 10.1007/s00248-026-02847-7 (PMC13755150; doi:10.1007/s00248-026-02847-7)
Supplement: Supplementary file 1 [file 248_2026_2847_MOESM1_ESM.docx]

Table A.1 – Measured sediment and water parameters from Cerastoderma edule sampling stations across three estuarine sites: TR (low impact), NAV (moderate to high impact), and GAM (intermediate impact), during Spring 2021 (Spr21) and Autumn 2020 (Aut20). Variables include granulometric fractions (Fine Fraction, Sand, Gravel), organic matter (OM), trace metal concentrations (Li, Mn, Ni, Cr, U, Ba, Mo, Co, Cu, Zn, As, Pb, Hg), carbon and nitrogen content (C_org_, N_org_), calcium carbonate (CaCO₃), and in situ water (Temperature, Salinity (Sal), Dissolved Oxygen (Oxy), and pH). Values are shown for each sampling point.

| Season | Location | Station | FineFraction (%) | Sand (%) | Gravel (%) | OM (%) | Li (mg/kg) | Mn (mg/kg) | Ni (mg/kg) | Cr (mg/kg) | U (mg/kg) | Ba (mg/kg) | Mo (mg/kg) |
| --- | --- | --- | --- | --- | --- | --- | --- | --- | --- | --- | --- | --- | --- |
| Aut20 | NAV | 1 | 28,283 | 66,954 | 4,764 | 2,252 | 27,362 | 99,691 | 15,719 | 26,666 | 1,900 | 259,062 | 3,440 |
|  |  | 2 | 25,130 | 72,178 | 2,692 | 1,786 | 27,164 | 99,403 | 21,687 | 25,062 | 1,937 | 257,906 | 3,414 |
|  |  | 3 | 52,110 | 46,360 | 1,530 | 3,187 | 37,845 | 135,201 | 18,805 | 38,064 | 2,844 | 250,198 | 5,899 |
|  | GAM | 1 | 13,592 | 68,760 | 17,647 | 0,984 | 25,902 | 69,018 | 11,426 | 17,556 | 1,538 | 264,675 | 1,801 |
|  |  | 2 | 13,556 | 70,925 | 15,518 | 0,828 | 23,431 | 89,725 | 253,115 | 429,682 | 1,157 | 237,014 | 8,964 |
|  |  | 3 | 20,158 | 66,540 | 13,301 | 0,744 | 27,645 | 93,254 | 15,591 | 26,343 | 1,522 | 254,848 | 1,527 |
|  | TR | 1 | 2,015 | 97,126 | 0,859 | 0,393 | 11,895 | 43,283 | 5,916 | 4,206 | 0,456 | 256,235 | 0,046 |
|  |  | 2 | 0,157 | 96,007 | 3,837 | 0,476 | 11,442 | 50,147 | 9,825 | 4,571 | 0,481 | 223,193 | 0,013 |
|  |  | 3 | 2,417 | 92,478 | 5,106 | 0,453 | 12,990 | 55,318 | 9,247 | 5,774 | 0,430 | 200,739 | 0,029 |
| Spr21 | NAV | 1 | 26,487 | 71,901 | 1,612 | 2,311 | 27,174 | 94,733 | 14,097 | 29,319 | 1,588 | 245,579 | 2,051 |
|  |  | 2 | 20,419 | 78,038 | 1,542 | 1,857 | 29,602 | 106,542 | 16,865 | 30,185 | 1,875 | 263,227 | 2,150 |
|  |  | 3 | 30,569 | 66,586 | 2,845 | 2,198 | 30,313 | 110,401 | 16,465 | 29,635 | 2,189 | 244,417 | 4,699 |
|  | GAM | 1 | 11,924 | 66,272 | 21,804 | 1,035 | 19,695 | 59,817 | 10,207 | 13,346 | 1,207 | 230,801 | 1,358 |
|  |  | 2 | 15,180 | 68,197 | 16,623 | 0,958 | 22,991 | 69,292 | 11,029 | 18,613 | 1,353 | 241,009 | 0,877 |
|  |  | 3 | 21,795 | 65,809 | 12,396 | 1,543 | 29,748 | 102,558 | 16,795 | 28,954 | 1,586 | 261,044 | 1,738 |
|  | TR | 1 | 3,754 | 94,913 | 1,333 | 0,600 | 14,031 | 50,811 | 7,796 | 6,719 | 0,501 | 262,873 | 0,064 |
|  |  | 2 | 3,049 | 84,301 | 12,650 | 0,449 | 13,627 | 60,949 | 8,270 | 6,640 | 0,599 | 206,240 | 0,023 |
|  |  | 3 | 2,144 | 95,524 | 2,332 | 0,470 | 12,937 | 46,507 | 12,357 | 4,979 | 0,468 | 247,236 | <LQ |

| Season | Location | Station | Co (mg/kg) | Cu (mg/kg) | Zn (mg/kg) | As (mg/kg) | Pb (mg/kg) | Hg (mg/kg) | C_org_ (%) | N_org_ (%) | CaCO_3_ (%) | Temp (°C) | Sal | Oxy (mg/L) | pH |
| --- | --- | --- | --- | --- | --- | --- | --- | --- | --- | --- | --- | --- | --- | --- | --- |
| Aut20 | NAV | 1 | 4,350 | 33,983 | 86,455 | 11,857 | 25,820 | 0,292 | 0,697 | 0,147 | 2,199 | 13,333 | 15,800 | 10,493 | 7,593 |
|  |  | 2 | 4,377 | 33,094 | 108,189 | 14,474 | 29,785 | 0,391 | 0,655 | 0,226 | 1,991 | 13,367 | 16,000 | 8,843 | 8,573 |
|  |  | 3 | 6,114 | 31,536 | 92,282 | 12,647 | 26,277 | 0,190 | 1,116 | 0,516 | 2,175 | 13,967 | 15,933 | 9,467 | 8,847 |
|  | GAM | 1 | 2,912 | 9,410 | 35,100 | 6,270 | 18,470 | 0,066 | 0,264 | 0,031 | 1,236 | 16,367 | 14,400 | 15,517 | 8,680 |
|  |  | 2 | 7,304 | 25,585 | 38,755 | 7,793 | 16,277 | 0,058 | 0,294 | 0,040 | 1,336 | 17,633 | 14,933 | 24,000 | 8,615 |
|  |  | 3 | 4,633 | 26,549 | 51,255 | 9,784 | 18,957 | 0,073 | 0,390 | 0,049 | 1,179 | 16,467 | 14,833 | 14,100 | 8,502 |
|  | TR | 1 | 0,702 | 2,231 | 5,266 | 2,024 | 14,085 | 0,015 | 0,103 | -0,026 | 2,890 | 17,300 | 14,767 | 14,567 | 8,107 |
|  |  | 2 | 0,694 | 1,627 | 5,460 | 1,759 | 11,883 | 0,028 | 0,134 | -0,089 | 3,731 | 16,933 | 15,800 | 14,033 | 8,167 |
|  |  | 3 | 0,885 | 3,464 | 13,008 | 1,043 | 11,322 | 0,033 | 0,133 | -0,125 | 4,051 | 16,200 | 13,567 | 13,167 | 7,974 |
| Spr21 | NAV | 1 | 4,570 | 45,924 | 127,330 | 10,837 | 31,479 | 0,283 | 0,564 | 0,055 | 1,356 | 23,133 | 14,333 | 4,267 | 7,496 |
|  |  | 2 | 4,890 | 43,585 | 141,015 | 14,444 | 35,373 | 0,468 | 0,631 | 0,057 | 1,696 | 22,300 | 16,200 | 7,000 | 8,045 |
|  |  | 3 | 4,512 | 24,086 | 59,499 | 8,789 | 21,206 | 0,181 | 0,900 | 0,082 | 1,922 | 26,233 | 14,367 | 4,733 | 8,002 |
|  | GAM | 1 | 2,366 | 7,900 | 28,370 | 3,671 | 15,712 | 0,075 | 0,169 | 0,021 | 1,238 | 23,633 | 16,433 | 8,333 | 8,473 |
|  |  | 2 | 3,533 | 12,119 | 40,682 | 7,524 | 16,815 | 0,070 | 0,292 | 0,036 | 1,437 | 23,933 | 15,600 | 8,233 | 7,850 |
|  |  | 3 | 5,089 | 21,450 | 57,109 | 9,365 | 19,728 | 0,117 | 0,409 | 0,044 | 1,268 | 23,300 | 10,100 | 9,733 | 8,054 |
|  | TR | 1 | 1,008 | 4,416 | 11,312 | 0,948 | 14,635 | 0,051 | 0,125 | 0,014 | 3,550 | 20,933 | 14,867 | 8,100 | 8,333 |
|  |  | 2 | 0,938 | 3,320 | 9,762 | 1,390 | 11,648 | 0,053 | 0,104 | -0,044 | 4,571 | 21,033 | 15,067 | 8,700 | 8,266 |
|  |  | 3 | 0,795 | 3,291 | 7,400 | 0,335 | 13,149 | 0,044 | 0,137 | -0,018 | 3,071 | 20,267 | 15,967 | 9,067 | 8,368 |

Table A.1 (continued)– Measured sediment and water parameters from Cerastoderma edule sampling stations across three estuarine sites: TR (low impact), NAV (moderate to high impact), and GAM (intermediate impact), during Spring 2021 (Spr21) and Autumn 2020 (Aut20). Variables include granulometric fractions (Fine Fraction, Sand, Gravel), organic matter (OM), trace metal concentrations (Li, Mn, Ni, Cr, U, Ba, Mo, Co, Cu, Zn, As, Pb, Hg), carbon and nitrogen content (C_org_, N_org_), calcium carbonate (CaCO₃), and in situ water parameters (Temperature, Salinity (Sal), Dissolved Oxygen (Oxy), and pH). Values are shown for each sampling point.

Table A.3 Alpha diversity of the gut microbiome of *Cerastoderma edule* for prokaryotes (16S rRNA gene) and fungi (ITS regions).

| Season | Location | 16S / ITS | Observed sp. | Shannon | Simpson | Chao1 |
| --- | --- | --- | --- | --- | --- | --- |
| Aut20 | GAM | 16S | 1116 | 8,50 | 0.99 | 1117,44 |
|  | NAV |  | 2224 | 8,74 | 0.988 | 2234,88 |
|  | TR |  | 593 | 2,71 | 0,68 | 594,99 |
|  | GAM | ITS | 151 | 3,44 | 0,826 | 151 |
|  | NAV |  | 154 | 1,45 | 0,350 | 154 |
|  | TR |  | 206 | 2,98 | 0,741 | 206 |
| Spr21 | GAM | 16S | 1667 | 7,26 | 0,963 | 1679,94 |
|  | NAV |  | 1575 | 6,27 | 0,886 | 1581,56 |
|  | TR |  | 707 | 3,63 | 0,699 | 712 |
|  | GAM | ITS | 71 | 2,93 | 0,825 | 71 |
|  | NAV |  | 71 | 2,91 | 0,825 | 71 |
|  | TR |  | 166 | 4,10 | 0,862 | 166 |
